# The accuracy and promise of personal breathalysers for research: Steps toward a cost-effective reliable measure of alcohol intoxication?

**DOI:** 10.1177/2055207617746752

**Published:** 2017-12-20

**Authors:** Benjamin C Riordan, Damian Scarf, Saleh Moradi, Jayde A M Flett, Kate B Carey, Tamlin S Conner

**Affiliations:** 1 Department of Psychology, 2495 University of Otago, New Zealand; 2 Department of Behavioral and Social Sciences, 118716 Center for Alcohol and Addiction Studies, Brown University School of Public Health, US

**Keywords:** BAC, personal breathalyser, alcohol use, technology

## Abstract

**Objective:**

Technology is continuing to shape the way we collect health data, including data on alcohol use. A number of technologies are being developed to objectively measure intoxication ‘in the wild’ without relying on self-report; the most immediate solution may be the use of personal breathalysers. In this study, we aimed to determine whether a cost-effective personal breathalyser would perform in a similar manner to a device used for roadside breath testing.

**Method:**

We intercepted young adults (*n* = 337; 45% men) outside three concerts, administered 5-min interviews, and asked for breath samples on two devices (a personal breathalyser and a police-grade breathalyser).

**Results:**

Participants reported having consumed an average of 7.3 standard drinks before the interview and had a mean Blood Alcohol Content of 0.077 g/dl on the police-grade device and 0.085 g/dl on the personal device. Difference scores suggested the personal breathalyser was more likely to over report Blood Alcohol Content (bias = 0.008 g/dl).

**Conclusion:**

Although the personal device was more likely to over report Blood Alcohol Content compared with the police-grade device, the results suggest that personal devices could be used as a measure of Blood Alcohol Content when collecting data outside of the lab.

## Introduction

Advances in technology continue to shape the way we collect health data. Rather than relying on participants’ self-report, emerging technologies offer a way to collect rich physiological and behavioral data in real-world environments.^1^ Recently, there has been a concerted effort to develop a device that can reliably measure alcohol intoxication, with the National Institute on Alcohol Abuse and Alcoholism (NIAAA) recently issuing its second Wearable Alcohol Biosensor Challenge.^2^ Despite the promise of wearable sensors (i.e. noninvasive devices that measure ethanol excreted through sweat),^3,4^ such devices are years away from reaching the levels of reliability required for research. There is, therefore, a need for other technologies to tide researchers over until new technology becomes available.

The most immediate and cost-effective solution toward collecting an objective measure of alcohol intoxication in the wild may be through the use of personal breathalysers (i.e. compact portable breathalysers marketed to the public). Since the last review,^5^ these devices are now able to sync to smartphones using Bluetooth, where readings can be stored, timestamped, and sent to researchers.^6^ Personal breathalysers provide an exciting avenue for alcohol research as they allow researchers to collect Blood Alcohol Content (BAC) estimates in naturalistic settings and to possibly sync data collection to tailored interventions delivered through smartphones *during* drinking sessions.^7–10^

Given recent advances in personal breathalysers, there is a need to determine whether this technology is accurate enough to be used in field studies for alcohol research. The aim of the present study was to determine whether a cost-effective personal breathalyser (BACtrack® Mobile Smartphone Breathalyser BT-M5) would offer similar readings and classifications to a ‘gold standard’ device used for roadside testing by law enforcement (LifeLoc® FC10; an Australian Standards certified breathalyser). We approached participants on three nights to provide breathalyser readings. We measured the differences in BAC between the devices and the extent to which the two devices would yield similar drunk driving classifications.

## Method

### Materials

The LifeLoc® FC10 is a police-grade device that retails for US$719, uses a fuel cell sensor, and requires recalibration every 12 months. It reports an accuracy range of ±0.005 BAC with scores up to 0.100 BAC, with a ±5% above .100 BAC.

The BACtrack® Mobile Smartphone Breathalyser ProBT-M5 syncs via Bluetooth to a mobile application and retails for US$99, uses a fuel cell sensor, and requires recalibration every 6–12 months. It does not offer an accuracy range.

Both devices recommend that for the most accurate results there should be 15 min between the last drink and the breath test.

### Procedure

The study was conducted on three nights during Orientation Week events (a period associated with a number of university-run concerts).^11^ Interviews were conducted directly outside the events most associated with alcohol use.^12,13^ The interviews were conducted by 10 trained researchers working in groups of two–three in the alcohol-free area outside events. Each group operated one of the four police-grade breathalysers, which provided Breath Alcohol Concentration (ug/L; converted to BAC, g/dl) and one of the five personal breathalysers (g/dl). The research groups approached participants, explained the purpose of the study, and invited them to take part in a 5-min interview. Those who agreed to take part provided verbal consent, answered questions about their drinking session, when they consumed their last drink, and provided a breath sample for both the police-grade and personal breathalyser (order of breathalysers was randomized). All study procedures were approved by the University of Otago Human Ethics Committee.

## Results

Of the 902 individuals approached, 337 were included in the main analysis. Individuals were excluded if they declined to take part (*n* = 145; 16%), had not consumed any alcohol (*n* = 99; 11%), had consumed alcohol within 10 min prior to the 5-min interview (*n* = 274; 30%), or if they did not provide a breath sample for both breathalysers (*n* = 47; 5%). The final sample was predominantly students (96%), about half were men (45%; 2% did not identify gender) who ranged in age from 17–28 (*M* = 18.4; *SD* = 1.5; two did not specify age). Participants reported consuming an average of 7.3 drinks (*CI* = 6.8, 7.7; *SD* = 4.4) and registered a BAC of 0.077 g/dl (*CI* = 0.072, 0.082; *SD* = 0.047) on the police-grade device, and 0.085 g/dl (*CI* = 0.079, 0.090; *SD* = 0.051) on the personal device.

### Bland–Altman test of agreement

In order to assess the amount of agreement between the two devices, we first calculated whether the difference between the two devices (bias) was significantly different from zero. When subtracting the police-grade breathalyser scores from the personal breathalyser scores, findings indicated that the personal device was more likely to over report BAC (bias = 0.008 g/dl, *CI* = 0.0062, 0.0096; *SD* = 0.015; *t*(337) = 9.578, *p* < 0.001; two-tailed).

**Figure 1.**
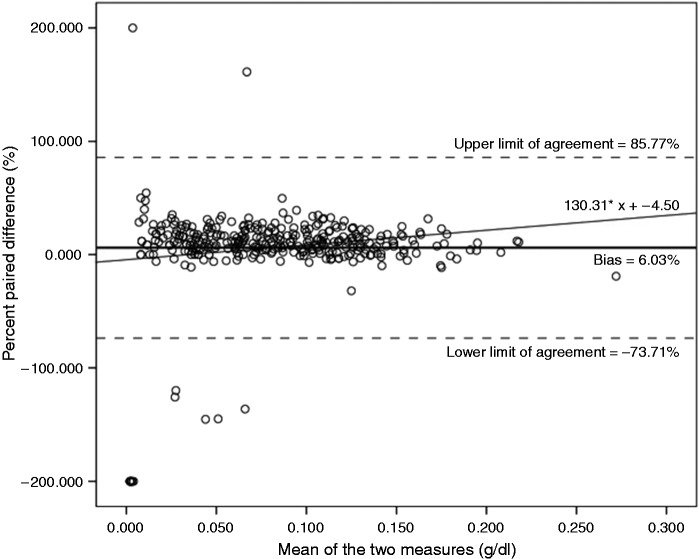
Difference in Blood Alcohol Content (BAC) for personal breathalysers vs. police-grade breathalysers for participants who had consumed no alcohol 10 min before the interview (*n* = 337). Positive values indicate over-estimation of BAC in personal breathalysers vs. police-grade breathalysers. Bold line indicates the mean of percent paired differences (bias = 6.03%), the upper 95% Limit of Agreement (upper LOA = 85.77%) and the lower 95% Limit of Agreement (lower LOA = −73.71%).

Next, we used a Bland and Altman (B&A) plot to describe the agreement between the devices.^14,15^ While there was a strong correlation between the two devices (*r*(337) = .955, *p < *.001), a strong correlation may not indicate good agreement. Therefore, in addition to the bias (0.008 g/dl), we calculated the lower and upper limits of agreement. Because the difference scores were not normally distributed (W = 0.792, *p < *.001),^16^ we used the percentage of difference between the measures.^17,18^ Following this approach, the calculated bias (i.e. the mean of percent paired differences) was 6.03%, *SD* = 40.69%, and the 95% limits of agreement were (bias −1.96*SD) = -73.71% and (bias +1.96*SD) = 85.77% (see Figure 1). While the bias of 6.03% suggests that the personal device consistently over reports, a closer look at the B&A plot seems to show greater bias at higher readings. Regressing the percent paired difference on the mean of the two measures somewhat supports this trend (*R^2^*^ ^= .024, *F*(1, 335) = 8.313, *p* = .004).

### Drunk driving classification

Finally, we used the drunk driving limit of 0.05 g/dl to determine whether the personal device would be similar in classification. Scores were recoded as 1 (at or over the 0.05 limit) or 0 (under the limit 0.000–0.049). As seen in Table 1, the sensitivity (proportion of true positives correctly identified) was 93.2% (*n* = 219) and the specificity (proportion of true negatives correctly identified) was 96.1% (*n* = 98).^17^ Thus, 94.1% (*n* = 313) of the time the two devices resulted in the same classification.

**Table 1.** Drunk driving classifications (+/− 0.05 g/dl) between the police-grade and the personal breathalyser.

		Police-grade		
		+0.05 g/dl	−0.05 g/dl	Total
Personal	+0.05 g/dl	219	16	235
	−0.05 g/dl	4	98	102
	Total	223	114	337

### Additional analyses

The interview took around 5 min, thus we retained those who had consumed alcohol more than 10 min before the interview began. But given that the interview varied in length, some participants may have been within the 15-min window recommended by manufacturers. For completeness, we present the results using a more conservative inclusion criterion in Supplementary materials (for those who had not consumed alcohol 15 min prior to the interview). There was very little difference between the two methods of inclusion.

## Discussion

The personal breathalyser was more likely to over report BAC (the mean of differences = 0.008 g/dl), particularly at higher levels. Although this bias may be an issue in clinical settings, the personal breathalyser may be a valid approach to collecting BAC outside of the lab.^19^ One could argue that the finding that the personal breathalyser was more likely to overestimate BAC is preferable, as it provides a margin of safety for individuals who use these devices to prevent drunk driving. Furthermore, the over report of BAC at higher readings is somewhat similar to inconsistencies that have been found between breath tests and formulas that predict BAC retrospectively.^20^

Regarding categorization, both devices effectively classified participants into similar drunk driving categories at a rate of 94.1%. With respect to research, given the limitations in retrospective reconstruction of BAC via self-report data,^20^ devices such as the BACtrack® may enhance the accuracy of field research on alcohol consumption.

### Limitations

The main limitation was the participants’ self-reported time since last drink. It is possible they overestimated the time and residual mouth alcohol could have tainted the breathalyser reports (this may account for some of the outliers on the B&A plot). It is also possible that some of the outliers may have been due to participants burping between breath tests, which can increase residual mouth alcohol and lead to inaccurate reports. Future research could supplement this study with tests in a more controlled environment. Although a controlled environment may be preferable,^5^ it is important for research to show how participants would interact with these devices in a natural setting.

### Considerations for use

Personal breathalysers may act in a similar manner to police-grade breathalysers, but several caveats are required. First, fuel cell devices such as the BACtrack® are preferable for research given that devices using semi-conductor sensors are significantly less accurate than police-grade devices.^6^ Second, there needs to be at least 15 min between the last drink and the breath sample, which presents some challenges for using these devices in naturalistic settings where drinking may be continuous.

## Conclusion

Devices such as the BACtrack® may be a promising tool to collect a measure of alcohol intoxication in naturalistic settings.

## Supplementary Material

Supplementary material

## References

[1] Conner TS and Mehl MR. Ambulatory assessment – methods for studying everyday life. In: Scott RA, Kosslyn SM and Pinkerton N (eds), *Emerging trends in the social and behavioral sciences*. Hoboken, NJ: Wiley, 2015, pp. 1–13.

[2] National Institute on Alcohol Abuse and Alcoholism (NIAAA), https://www.niaaa.nih.gov/challenge-prize (2017, accessed 31 January 2017).

[3] Barnett NP Tidey J Murphy JG et al. Contingency management for alcohol use reduction: a pilot study using a transdermal alcohol sensor. Drug Alcohol Depen 2007; 118: 391–399. 10.1016/j.drugalcdep.2011.04.023 PMC3190068 21665385

[4] Karns-Wright TE Roache JD Hill-Kapturczak N et al. Time delays in transdermal alcohol concentrations relative to breath alcohol concentrations. Alcohol Alcoholism 2017; 52: 35–41. 27522029 10.1093/alcalc/agw058 PMC5169032

[5] Ashdown HF Fleming S Spencer EA et al. Diagnostic accuracy study of three alcohol breathalysers marketed for sale to the public. BMJ Open 2014; 4: e005811. 10.1136/bmjopen-2014-005811 PMC4281544 25526794

[6] BACtrack, https://www.bactrack.com/collections/smartphone-breathalyzers (2017, accessed 31 January 2017).

[7] Renner KA Walker N Parag V et al. Harm reduction text messages delivered during alcohol drinking: feasibility study protocol. JMIR Res Protoc 2012; 1: e4. 23611773 10.2196/resprot.1970 PMC3626143

[8] Riordan BC Conner TS Flett JA et al. A brief orientation week ecological momentary intervention to reduce university student alcohol consumption. J Stud Alcohol Drug 2015; 76: 525–529. 10.15288/jsad.2015.76.525 26098027

[9] Wright CJ Dietze PM Crockett B et al. Participatory development of MIDY (Mobile Intervention for Drinking in Young people). BMC Public Health 2016; 16: 184. 26911299 10.1186/s12889-016-2876-5 PMC4765036

[10] Riordan BC, Conner TS, Flett JA, et al. A text message intervention to reduce first year university students’ alcohol use: a pilot experimental study. *Digital Health* 2017; 3: 1–10. 10.1177/2055207617707627 PMC6001236 29942597

[11] Riordan BC Scarf D Conner TS Is orientation week a gateway to persistent alcohol use in university students? A preliminary investigation. J Stud Alcohol Drugs 2015; 76: 204–211. 25785795 10.15288/jsad.2015.76.204

[12] Riordan BC Flett JA Conner TS et al. Text message interventions for alcohol use: current research and future directions. In: Gutierres W (ed). Alcohol consumption: Patterns, influences, and health effects, New York, NY: Nova, 2016, pp. 185–192.

[13] Riordan BC, Conner TS, Flett JA, et al. An intercept study to measure the extent to which New Zealand university students pre-game. *Aust NZ J Public Health.* Forthcoming 2017. 10.1111/1753-6405.12754 29281165

[14] Bland JM Altman D Statistical methods for assessing agreement between two methods of clinical measurement. Lancet 1986; 327: 307–310. 2868172

[15] Altman DG Bland JM Diagnostic tests. 1: Sensitivity and specificity. Brit Med J 1994; 308: 1552. 8019315 10.1136/bmj.308.6943.1552 PMC2540489

[16] Shapiro SS Wilk MB An analysis of variance test for normality (complete samples). Biometrika 1965; 52: 591–611.

[17] Bland JM Altman DG Applying the right statistics: analyses of measurement studies. Ultrasound Obst Gyn 2003; 22: 85–93. 10.1002/uog.122 12858311

[18] Linnet K Bruunshuus I HPLC with enzymatic detection as a candidate reference method for serum creatinine. Clin Chem 1991; 37: 1669–1675. 1914163

[19] Andersson AK Kron J Castren M et al. Assessment of the breath alcohol concentration in emergency care patients with different levels of consciousness. Scand J Trauma Resusc Emerg Med 2015; 23: 11. 25652597 10.1186/s13049-014-0082-y PMC4332718

[20] Hustad JT Carey KB Using calculations to estimate blood alcohol concentrations for naturally occurring drinking episodes: a validity study. J Stud Alcohol Drug 2005; 66: 130–138. 10.15288/jsa.2005.66.130 15830913

