# Supplementary material for: The accuracy and promise of personal breathalysers for research: Steps toward a cost-effective reliable measure of alcohol intoxication?
Source: Digit Health. 2017 Dec 20;3:2055207617746752. doi: 10.1177/2055207617746752 (PMC6001255; doi:10.1177/2055207617746752)
Supplement: Supplementary material [file Supplementary_Table_1_and_Figure_1.pdf]

Supplementary Table 1. Relation between drunk driving (+/- 0.05g/dl) between the police-grade and the personal breathalyser for participants who had consumed no alcohol within 15 minutes of the interview (*n* = 258).

		**Police-Grade**		
		+0.05g/dl	-0.05g/dl	**Total**
**Personal**	+0.05g/dl	170	12	182
	-0.05g/dl	1	75	76
	**Total**	171	87	258

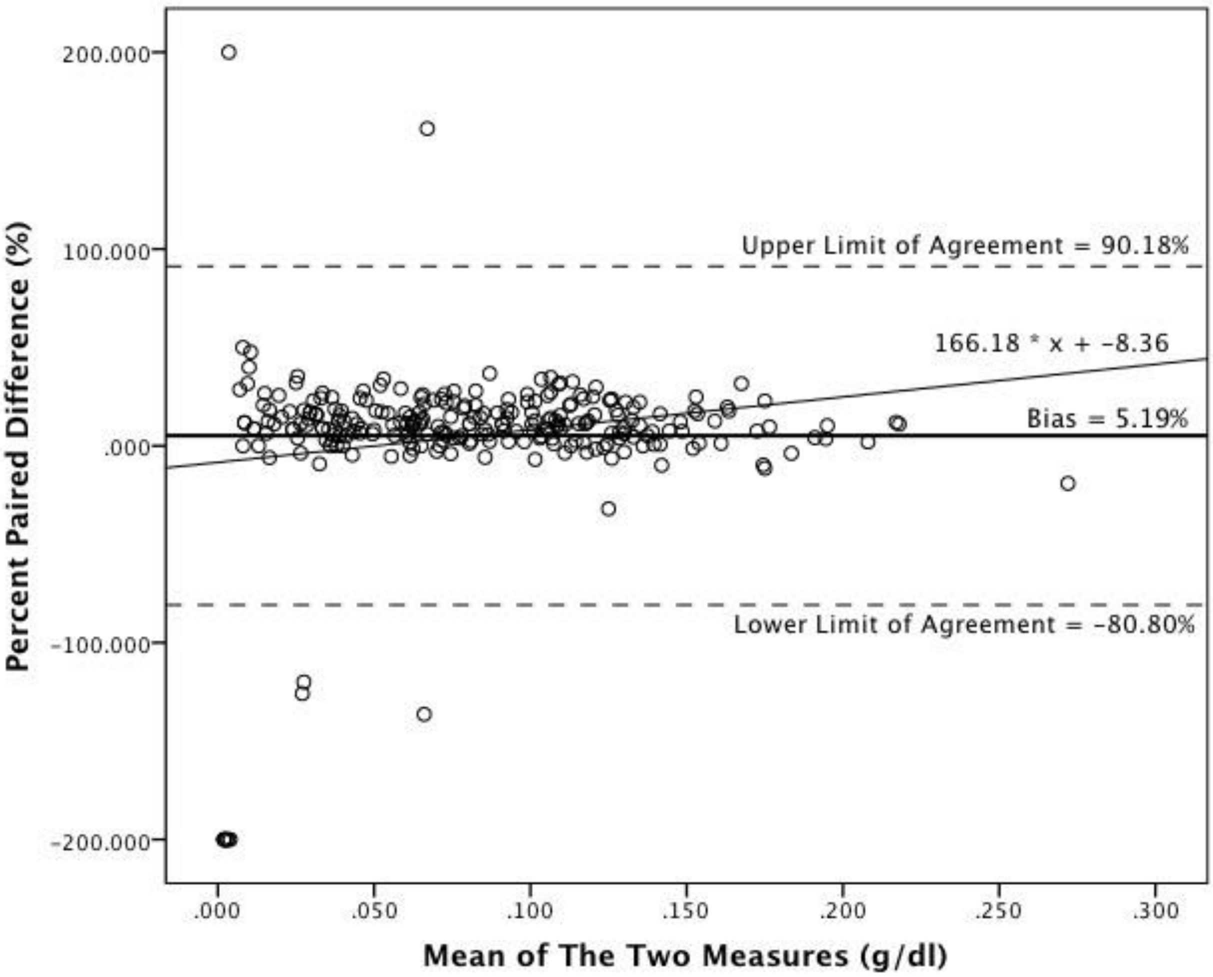

Upper Limit of Agreement = 90.18%
166.18 * x + -8.36
Bias = 5.19%
Lower Limit of Agreement = -80.80%
200.000
100.000
.000
-100.000
-200.000
.000
.050
.100
.150
.200
.250
.300
Percent Paired Difference (%)
Mean of The Two Measures (g/dl)
